# Generation of chromosome 1p/19q co-deletion by CRISPR/Cas9-guided genomic editing

**DOI:** 10.1093/noajnl/vdac131

**Published:** 2022-08-18

**Authors:** Chao Li, Zhong Liu, Xiaoxia Zhang, Huafeng Wang, Gregory K Friedman, Qiang Ding, Xinyang Zhao, Hu Li, Kitai Kim, Xi Yu, L Burt Nabors, Xiaosi Han, Rui Zhao

**Affiliations:** Department of Biochemistry and Molecular Genetics, University of Alabama at Birmingham, AL 35294, USA; Department of Biochemistry and Molecular Genetics, University of Alabama at Birmingham, AL 35294, USA; Department of Biochemistry and Molecular Genetics, University of Alabama at Birmingham, AL 35294, USA; Department of Genetics, University of Alabama at Birmingham, AL 35294, USA; Department of Neurology, University of Alabama at Birmingham, AL 35294, USA; Department of Pediatrics, Division of Hematology/Oncology, University of Alabama at Birmingham, Birmingham, AL 35294, USA; Department of Anesthesiology and Perioperative Medicine & Molecular and Translational Biomedicine, University of Alabama at Birmingham, Birmingham, AL 35294, USA; Department of Biochemistry and Molecular Genetics, University of Alabama at Birmingham, AL 35294, USA; Department of Molecular Pharmacology & Experimental Therapeutics, Center for Individualized Medicine, Mayo Clinic College of Medicine, Rochester, MN 55904, USA; Human Stem Cell & Genome Engineering Center and Department of Biological Chemistry, University of California, Los Angeles, CA 90095, USA; Clinical Oncology Center, The People’s Hospital of Guangxi Zhuang Autonomous Region, Nanning, Guangxi 530021, China; Department of Neurology, University of Alabama at Birmingham, AL 35294, USA; Department of Neurology, University of Alabama at Birmingham, AL 35294, USA; Department of Biochemistry and Molecular Genetics, University of Alabama at Birmingham, AL 35294, USA; Gregory Fleming James Cystic Fibrosis Research Center, University of Alabama at Birmingham, Birmingham, AL 35294, USA

**Keywords:** chromosome translocation, CRISPR/Cas9, *IDH* mutant low-grade gliomas, oligodendroglioma, 1p/19q co-deletion

## Abstract

**Background:**

Chromosomal translocation has been detected in many human cancers including gliomas and is considered a driving force in tumorigenesis. Co-deletion of chromosome arms 1p and 19q is a hallmark for oligodendrogliomas. On the molecular level, 1p/19q co-deletion results from t(1;19)(q10;p10), which leads to the concomitant formation of a hybrid chromosome containing the 1q and 19p arms. A method to generate 1p/19q co-deletion is lacking, which hinders the investigation of how 1p/19q co-deletion contributes to gliomagenesis.

**Methods:**

We hypothesized that chromosomal translocation, such as t(1;19)(q10;p10) resulting in the 1p/19q co-deletion, may be induced by simultaneously introducing DNA double-strand breaks (DSBs) into chromosomes 1p and 19q using CRISPR/Cas9. We developed a CRISPR/Cas9-based strategy to induce t(1;19)(q10;p10) and droplet digital PCR (ddPCR) assays to detect the hybrid 1q/19p and 1p/19q chromosomes.

**Results:**

After translocation induction, we detected both 1p/19q and 1q/19p hybrid chromosomes by PCR amplification of the junction regions in HEK 293T, and U-251 and LN-229 glioblastoma cells. Sequencing analyses of the PCR products confirmed DNA sequences matching both chromosomes 1 and 19. Furthermore, the 1p/19q hybrid chromosome was rapidly lost in all tested cell lines. The 1q/19p hybrid chromosome also become undetectable over time likely due to cell survival disadvantage.

**Conclusion:**

We demonstrated that t(1;19)(q10;p10) may be induced by CRISPR/Cas9-mediated genomic editing. This method represents an important step toward engineering the 1p/19q co-deletion to model oligodendrogliomas. This method may also be generalizable to engineering other cancer-relevant translocations, which may facilitate the understanding of translocation roles in cancer progression.

Key Points

- Induction of 1p/19q co-deletion by CRISPR/Cas9-guided genomic editing.
- This method is an important step toward engineering a model for oligodendrogliomas.

**Importance of the Study**

Co-deletion of chromosome arms 1p and 19q, which results from chromosomal translocation, is a hallmark for oligodendrogliomas. How 1p/19q co-deletion contributes to gliomagenesis and cooperates with other glioma-relevant mutations remains largely unknown, primarily because recapitulating translocation events in cancer cells is challenging. In this study, we developed a CRISPR/Cas9-based strategy to induce t(1;19)(q10;p10) and the subsequent 1p/19q co-deletion in glioma cells. This method represents a critical step to developing a genetically defined research model for oligodendrogliomas to facilitate glioma research and the discovery of potential cures. This method may also be generalizable to engineering other cancer-relevant translocations, which may facilitate the understanding of translocation roles in cancer progression.

Chromosomal translocation has been detected in many human cancers including gliomas.^1,2^ As a multistep process, chromosomal translocation involves concomitant DNA double-strand breaks (DSBs) in multiple genomic locations and the joining of DNA ends of heterologous chromosomes by the cellular DNA repair mechanisms.^3^ Chromosomal translocation is often considered a driving force in tumorigenesis because it may lead to the formation of a fusion oncogene, misregulation of a proto-oncogene, disruption of a tumor suppressor gene, and/or loss of large chromosomal regions containing hundreds or even thousands of genes.^3^ However, the exact biological consequence resulting from a chromosomal translocation in tumor cells is generally difficult to investigate, primarily because recapitulating translocation events in cancer cells is challenging.

Co-deletion of chromosome arms 1p and 19q is frequently observed in gliomas with oligodendroglial features,^4,5^ such as low-grade oligodendrogliomas (WHO grade II) and anaplastic oligodendroglial tumors (WHO grade III).^6–9^ On a molecular level, co-deletion of the entire 1p and 19q arms results from a chromosomal translocation event (ie, t(1;19)(q10;p10)), which also leads to the concomitant formation of a hybrid chromosome containing the 1q and 19p arms.^10,11^ Interestingly, the 1p/19q co-deletion mostly occurs in oligodendrogliomas that also carry mutations in isocitrate dehydrogenase genes (ie, *IDH1* or *IDH2*), promoter of the telomerase gene (ie, *TERT*), and mutations in genes such as *CIC* or *FUBP1*.^12–14^ Although 1p/19q co-deletion has been reliably used as a diagnostic marker for oligodendrogliomas,^15,16^ how it contributes to gliomagenesis and cooperates with *IDH* and other mutations remains largely unknown. As an initial step toward creating a genetically defined oligodendroglioma model, we investigated how to engineer the translocation and subsequent 1p/19q co-deletion in glioma cells.

In this study, we developed a CRISPR/Cas9-based approach to induce t(1;19)(q10;p10) in glioblastoma (GBM) cell lines. We detected the formation of the two hybrid chromosomes that contain the 1p/19q arms and the 1q/19p arms with subsequent rapid loss of the 1p/19q hybrid chromosome but longer retention of the 1q/19p hybrid chromosome. This method is an important step toward engineering the 1p/19q co-deletion to model human oligodendrogliomas. Furthermore, this method may be generalizable to engineering other cancer-relevant chromosomal translocations and deletions, which are critical to understanding the causal roles of translocation in cancer progression.

## Materials and Methods

### Ethics Statement

This study is Non-Human Subject Research (NHSR) and does not involve laboratory animals. This study was approved by the Institutional Review Board (IRB) and Research Safety Committee of University of Alabama at Birmingham (UAB).

### Cell Culture

HEK 293T cells and GBM cell lines LN-229 and U-251 were obtained from American Type Culture Collection (ATCC).^17^ HEK 293T cells were cultured in DMEM (Gibco) with 10% fetal bovine serum (FBS; GeminiBio). The GBM cell lines were cultured in DMEM/F12 (Gibco) supplemented with 10% FBS and GlutaMax (Gibco) as described.^18^ All cell cultures were maintained at 37°C, 20.8% O_2_, and 5% CO_2_.

### CRISPR/Cas9-mediated Genomic Editing

Guide RNAs (gRNAs) recognizing chromosomes 1p and 19q sequences were designed using the CRISPick website (https://portals.broadinstitute.org/gppx/crispick/public). Two gRNAs for each chromosome arm were chemically synthesized (Synthego) (Supplementary Table S1). Nucleofection of ribonucleoprotein (RNP) complexes consisting of gRNAs and Cas9 was performed as described with modifications.^19^ In brief, 400 pmol of HiFi Cas9 (Integrated DNA Technologies) and 500 pmol sgRNA were mixed and incubated at room temperature for 10 minutes. 1 × 10^6^ cells were washed with Opti-MEM (ThermoFisher Scientific) and resuspended in 100 μl Nucleofector Solution (Lonza), gently mixed with pre-assembled RNPs, and electroporated with appropriate programs using Nucleofector 2b or 4d (Lonza). The nucleofection program used for HEK 293T, LN-229, and U-251 cells was Q-001, X-009, and DS-138, respectively.

### Genomic DNA Extraction, PCR, and Automatic Sequencing

Genomic DNA samples were prepared using the Quick-DNA Miniprep Kit (Zymo Research) or DNeasy Blood and Tissue Kit (Qiagen) as described.^20^ A nested PCR protocol consisting of two rounds of PCR reactions was performed to amplify the 1p/19q and the 1q/19p translocation junction fragments. Each PCR reaction of the first round contained 6.25 μl 2× LA PCR Mix (Takara Bio), 1 μl forward and reverse primer mix (20 μM), and ~ 200 ng genomic DNA template. PCR reaction was performed on a Mastercycler Nexus PCR Thermal Cycler (Eppendorf) using a thermal cycling program consisting of 20 cycles of denaturation at 95°C for 15 s, annealing at 57°C for 15 s, and elongation at 68°C for 30 s. The PCR products were diluted 100× and used as the template in round 2. Each PCR reaction of the second round contained 12.5 μl 2× LA PCR mix (Takara Bio), 2 μl nested forward and reverse primer mix (20 μM), and 1 μl diluted round 1 product. The thermal cycling program of the round 2 reaction consisted of 40 cycles of denaturation at 95°C for 15 s, annealing at 57°C for 15 s, and elongation at 68°C for 30 s. PCR products were purified using the PCR purification kit (Qiagen) before automatic sequencing. DNA sequences were viewed and analyzed by the SnapGene software (Dotmatics). Sequences of all primers are listed in Supplementary Table S2. Primer combinations for nested PCR reactions and sequencing analyses are listed in Supplementary Table S3.

### Droplet Digital PCR Analysis

Droplet digital PCR was performed as described.^21^ In brief, each reaction contained 11 μl 2x ddPCR Supermix for Probes (Bio-Rad), 2 μl target forward and reverse primer mix (20 μM), 1 μl target TaqMan Probe (FAM, 5 μM), 2 μl CCR5 internal control forward and reverse primer mix (20 μM), 1 μl CCR5 internal control TaqMan Probe (HEX, 5 μM), 100 ng genomic DNA, and water to a final volume of 22 μl. Droplets were generated on a QX200 Droplet Generator per the manufacturer’s instruction (Bio-Rad). PCR reactions were performed on a Mastercycler Nexus PCR Thermal Cycler (Eppendorf) using a thermal cycling program consisting of 40 cycles of denaturation at 95°C for 30 s and annealing/elongation at 57°C for 1 min. The droplets were then detected and analyzed by a QX200 Droplet Reader (Bio-Rad). Sequences of all primers and probes are listed in Supplementary Table S2. Primer and probe combinations for ddPCR reactions are listed in Supplementary Table S3.

## Results

### Chromosomal Translocation Resulting in the 1p/19q Co-deletion can be induced by CRISPR/Cas9

**Figure 1:**
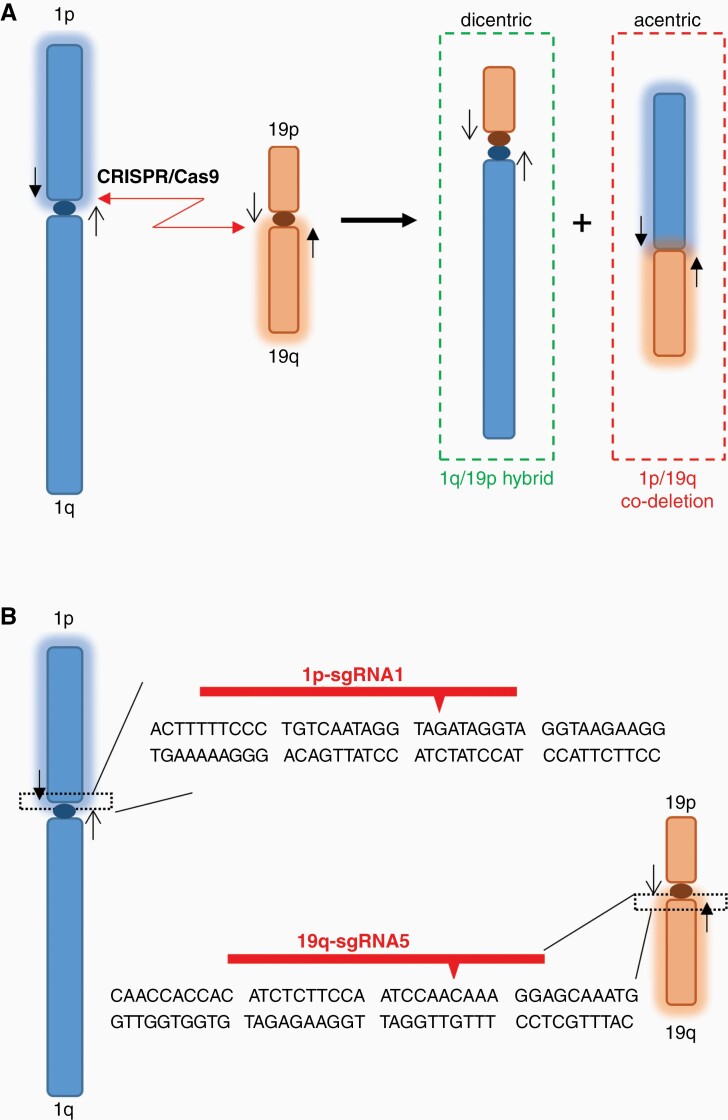
**CRISPR/Cas9-mediated chromosomal translocation and 1p/19q co-deletion. (A)** Schematic to illustrate the CRISPR/Cas9-mediated translocation and the subsequent 1p/19q co-deletion. **(B)** gRNAs introducing DNA double-stranded breaks (DSBs) in 1p and 19q. Red arrowheads, Cas9 cutting sites; black arrows, primer pairs encompassing the expected DSB sites on chromosomes 1 and 19.

Chromosomal translocation involves concomitant DSBs at multiple genomic loci and the subsequent joining of free DNA ends by the cellular DNA repair machinery.^3^ Because the CRISPR/Cas9 system can introduce DSBs at specific genomic loci, which are often repaired by the nonhomologous end joining (NHEJ) pathway,^22–24^ we hypothesize that chromosomal translocation, such as the t(1;19)(q10;p10) translocation resulting in the 1p/19q co-deletion in oligodendrogliomas,^10,11^ may be induced by simultaneously introducing DSBs into chromosomes 1p and 19q using CRISPR/Cas9 (Figure 1A). We expected the formation of a dicentric hybrid chromosome containing the 1q/19p arms and an acentric 1p/19q product. Because the acentric 1p/19q chromosome lacks a functional centromere, we anticipated it would be lost in dividing cells (Figure 1A).

To test this hypothesis, we designed gRNAs specifically recognizing nonrepetitive DNA sequences close to the centromeres (ie, within the chromosome 1p10 and 19q10 regions). Translocation induced by these gRNAs is expected to delete the entire 1p and 19q arms because of lacking a functional centromere in the 1p/19q product (Figure 1B). Two independent gRNAs to target either 1p or 19q were designed and chemically synthesized (Supplementary Table S1). Ribonucleoprotein (RNP) complexes containing gRNAs and Cas9 protein were assembled *in vitro* and introduced into HEK 293T cells. We chose HEK 293T cells to test genomic editing efficiencies and develop detection assays, primarily because these cells are easy to culture and transfect, as shown in previous studies.^22,23^ Cells prefer to repair DSBs by the NHEJ pathway, which frequently introduces insertions and deletions (INDELs) of several nucleotides at the repair sites.^25^ Therefore, the efficiency of each gRNA in instructing Cas9 to cut its DNA target can be roughly estimated by the INDEL frequencies, which can be visualized as mixed sequencing signals downstream of the cleavage site in the chromatograms of automatic DNA sequencing analyses (Supplementary Figure S1). Our data demonstrated all tested gRNAs effectively cut the target DNA sequences in chromosome 1p or 19q (Supplementary Figure S1). We chose one gRNA to target each chromosome arm (ie, 1p-sgRNA1 and 19q-sgRNA5) for further investigation (Figure 1B).

**Figure 2:**
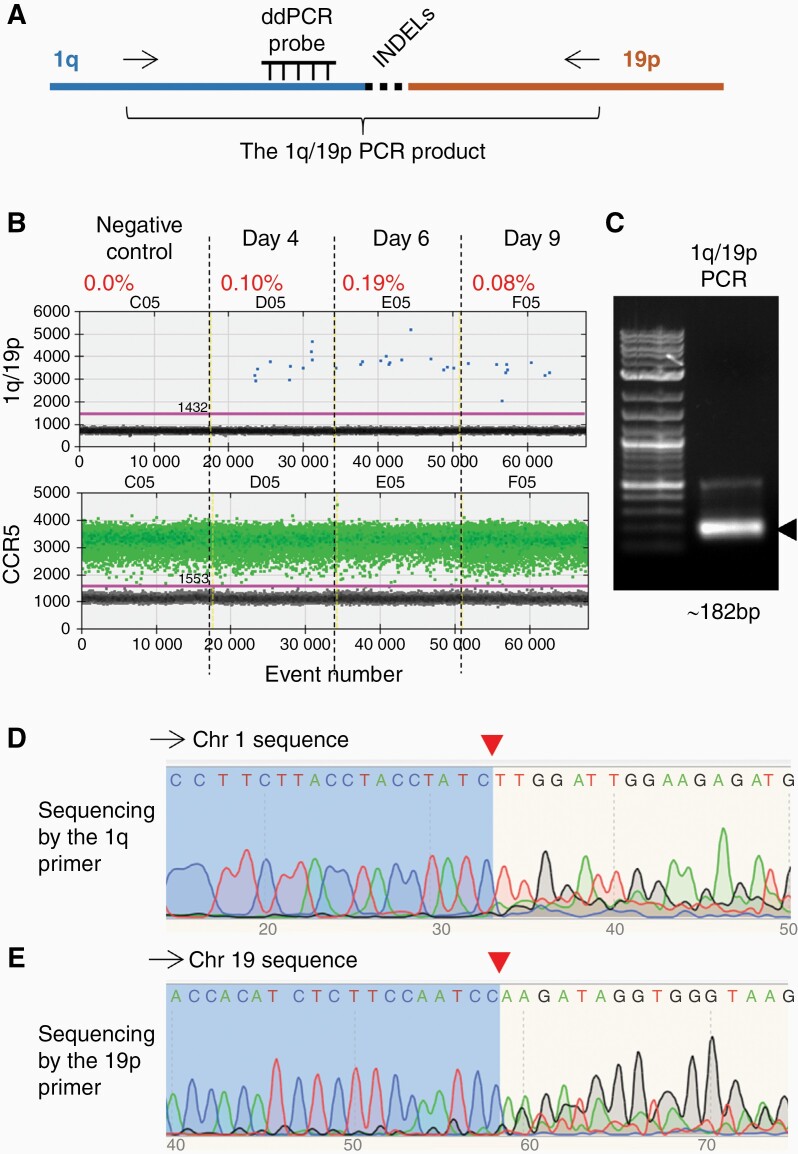
**Detection of the hybrid chromosome containing the 1q/19p arms. (A)** Schematic of the PCR assay that specifically detects the hybrid chromosome containing the 1q/19p arms (the 1q/19p product). To ensure reaction specificity, primer pairs recognizing 1q and 19p (arrows) and an internal Taqman probe recognizing 1q sequences have been used. INDELs, insertions and deletions of several nucleotides at the DSB repair site. Note that the internal probe is designed to recognize sequences outside the anticipated Cas9-cutting site and INDEL region. **(B)** Droplet digital PCR (ddPCR) analysis of the 1q/19p product in HEK 293T cells collected on different days after CRISPR/Cas9 introduction. CCR5, internal control to normalize the copy number of input genomic DNA. The percentage shows the fraction of 1q/19p positive droplets of each sample. **(C)** Electrophoresis of the 1q/19p product. Arrow, the expected PCR product. DNA ladder, 1KB Plus (NEB #N3200S). **(D-E)** Automatic sequencing analysis of the 1q/19p product using **(D)** the 1q primer and **(E)** the 19p primer. Red arrowheads, Cas9 cutting sites.

**Figure 3:**
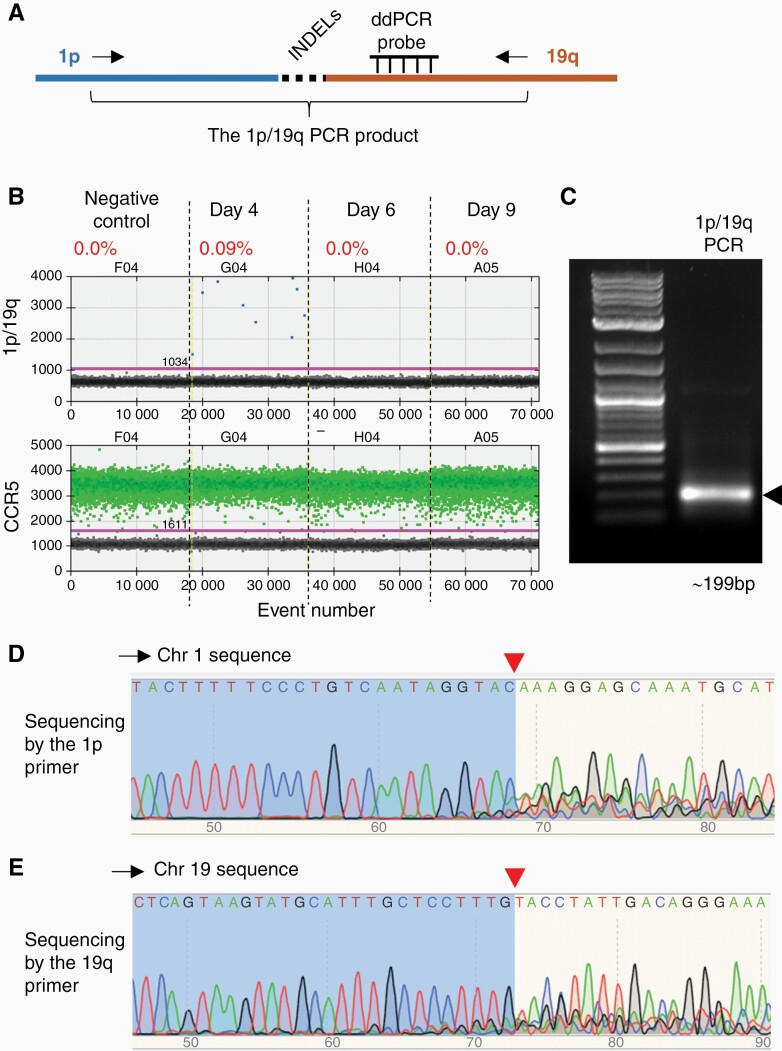
**Detection of the hybrid chromosome containing the 1p/19q arms. (A)** Schematic of the PCR assay that specifically detects the hybrid chromosome containing the 1p/19q arms (the 1p/19q product). To ensure reaction specificity, primer pairs recognizing 1p and 19q (arrows) and an internal Taqman probe recognizing 19q sequences have been used. INDELs, insertions and deletions of several nucleotides at the DSB repair site. Note that the internal probe is designed to recognize sequences outside the anticipated Cas9-cutting site and INDEL region. **(B)** Droplet digital PCR (ddPCR) analysis of the 1p/19q product in HEK 293T cells collected on different days after CRISPR/Cas9 introduction. CCR5, internal control to normalize the copy number of input genomic DNA. The percentage shows the fraction of 1p/19q positive droplets of each sample. **(C)** Electrophoresis of the 1p/19q product. Arrow, the expected PCR product. DNA ladder, 1KB Plus (NEB #N3200S). **(D-E)** Automatic sequencing analysis of the 1p/19q product using **(D)** the 1p primer and **(E)** the 19q primer. Red arrowheads, Cas9 cutting sites.

To induce the translocation and 1p/19q co-deletion, we simultaneously introduced RNPs containing gRNAs targeting 1p and 19q into HEK 293T cells (Figures 2 and 3). Genomic DNA samples were collected 4, 6, and 9 days after RNP introduction. To detect the translocation, we developed droplet digital PCR (ddPCR) assays to specifically detect the DNA junctions resulting from joining the DNA ends of 1q and 19p arms (ie, the 1q/19p product) and the 1p and 19q arms (ie, the 1p/19q product) (Figures 2A and 3A).

As expected, no 1q/19p or 1p/19q products were detected in DNA samples prepared from HEK 293T cells without RNP introduction (the negative controls) (Figures 2B and 3B). However, we were able to detect both the 1q/19p and 1p/19q products in DNA samples prepared 4 days after the RNP introduction at frequencies of approximately 0.1% (Figures 2B and 3B). Intriguingly, the 1q/19p product, which represents the dicentric hybrid chromosome containing the 1q and 19p arms (Figure 1A), was retained in cells at a relatively stable frequency at least 9 days after the RNP introduction (Figure 2B), while the 1p/19q product, which represents the acentric 1p/19q hybrid, was undetectable 6 days after RNP introduction (Figure 3B), demonstrating a rapid loss of the 1p and 19q arms.

To further confirm that CRISPR/Cas9 has induced translocation between chromosomes 1 and 19, we amplified and sequenced the 1q/19p and 1p/19q PCR products (Figures 2C and 3C). The PCR products were sequenced from both ends, using primers recognizing sequences in chromosomes 1 and 19, respectively (Supplementary Table S2 and S3). When sequencing with primers from chromosome 1, the sequence trace of readable, discrete peaks matching chromosome 1 were detected up to the Cas9 cutting site. The sequence trace downstream of the Cas9 cutting site contained overlapping and superimposed peaks because of INDELs introduced by NHEJ (Figures 2D and 3D). Conversely, when sequencing with primers from chromosome 19, clean DNA sequences matching chromosome 19 were detected up to the Cas9 cutting site. The sequence trace downstream of the Cas9 cutting site contained overlapping and superimposed peaks due to INDELs (Figures 2E and 3E). Together, these data demonstrate that chromosome fusion containing the 1q/19p arms and 1p/19q arms were formed in a fraction of HEK 293T cells (~0.1%) as a consequence of repairing CRISPR/Cas9-induced DSBs, and the acentric 1p/19q hybrid chromosome was rapidly lost.

### Induced t(1;19)(q10;p10) Translocation and 1p/19q Co-deletion in Glioma Cell Lines

**Figure 4:**
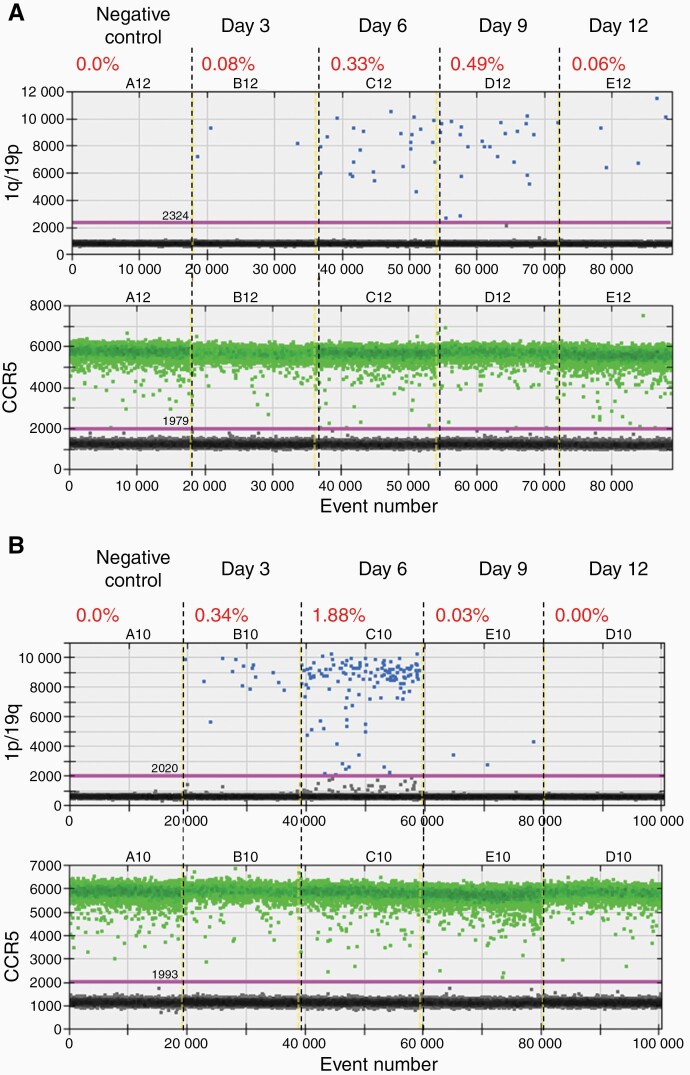
**CRISPR/Cas9-induced chromosomal translocation and 1p/19q co-deletion in the U-251 glioblastoma cells. (A)** Droplet digital PCR (ddPCR) analysis of the 1q/19p product in U-251 cells collected on different days after CRISPR/Cas9 introduction. **(B)** ddPCR analysis of the 1p/19q product in U-251 cells collected on different days after CRISPR/Cas9 introduction. CCR5, internal control to normalize the copy number of input genomic DNA. The percentage shows the fraction of 1q/19p or 1p/19q positive droplets of each sample.

**Figure 5:**
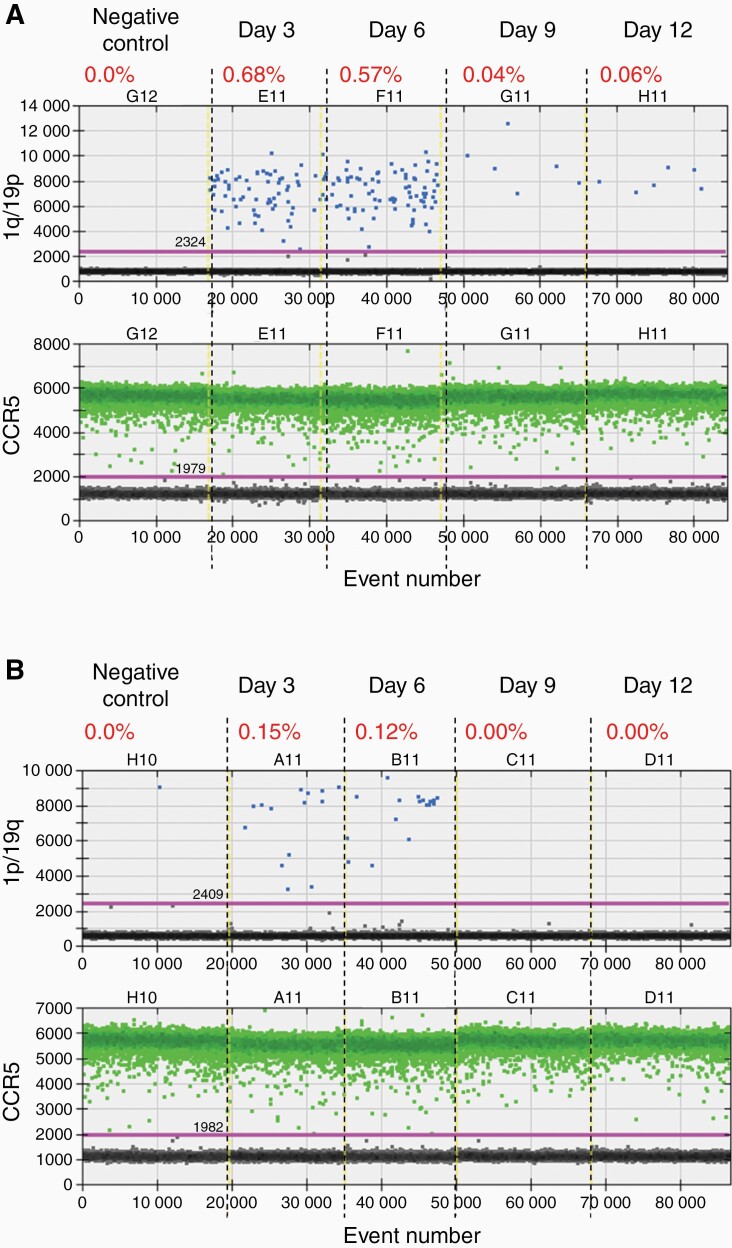
**CRISPR/Cas9-induced chromosomal translocation and 1p/19q co-deletion in the LN-229 glioblastoma cells. (A)** Droplet digital PCR (ddPCR) analysis of the 1q/19p product in LN-229 cells collected on different days after CRISPR/Cas9 introduction. **(B)** ddPCR analysis of the 1p/19q product in LN-229 cells collected on different days after CRISPR/Cas9 introduction. CCR5, internal control to normalize the copy number of input genomic DNA. The percentage shows the fraction of 1q/19p or 1p/19q positive droplets of each sample.

Next, we investigated whether this method of inducing translocation and 1p/19q co-deletion was generalizable to other more disease-relevant cell lines such as GBM U-251 and LN-229 cells.^17^ Using a similar approach, we introduced RNPs containing the gRNAs targeting chromosome 1p and 19q in U-251 and LN-229 cells. We collected genomic DNA from the cells 3, 6, 9, and 12 days after the RNP introduction and performed ddPCR to detect the 1q/19p and 1p/19q fusion products (Figures 4 and 5).

As expected, neither 1q/19p nor 1p/19q signals were detected in U-251 or LN-229 cells without RNP introduction (the negative controls) (Figures 4 and 5), indicating that these GBM cells do not contain the hybrid chromosomes. Interestingly, the 1q/19p and 1p/19q products were detected in both U-251 and LN-229 cells with RNP introduction (Figures 4 and 5). In U-251 cells, the 1q/19p and 1p/19q signals were readily detected 3 days after RNP introduction (Figure 4A-B). The 1q/19p signal remained relatively stable from day 6 to day 9 (~0.3–0.5%) and decreased to 0.06% by day 12 (Figure 4A). However, the 1p/19q signal peaked on day 6 at a frequency of 1.88%, rapidly lost the signal to 0.03% on day 9, and was nondetectable on day 12 (Figure 4B). In LN-229 cells, the 1q/19p and 1p/19q products were detected 3 days after RNP introduction at a frequency of 0.68% and 0.15%, respectively (Figure 5A-B). While both decreased significantly 9 days after the RNP introduction, the 1q/19p signal was still observed at a frequency of 0.06% by day 12 whereas the 1p/19q signal became undetectable by day 9 (Figure 5A-B). Together, these data demonstrated that t(1;19)(q10;p10) has been induced by CRISPR/Cas9 in both tested GBM cell lines, and the acentric 1p/19q hybrid chromosome is quickly lost in both cases.

Next, we determined whether GBM cells carrying the 1q/19p hybrid chromosome could be isolated and expanded. To address this, we first examined whether the 1q/19p hybrid chromosome could be stably maintained by a small fraction of GBM cells. We extended the duration of our experiments and conducted ddPCR analyses on the 1q/19p fusion product (Supplementary Figure S2). We focused on the LN-229 cells, because they showed a higher 1q/19p translocation efficiency than U-251 cells (Figures 4A and 5A). We found that the 1q/19p signal was reduced to the background level 18 days after RNP introduction (Supplementary Figure S2), suggesting that GBM cells carrying the 1q/19p hybrid chromosome failed to proliferate and got lost over time in culture.

## Discussion

Recurrent chromosomal translocations, which generate mutations and lead to major genome rearrangement, are hallmarks of many human cancers.^26,27^ How translocations contribute to cancer is better understood when a specific translocation event leads to activity change of a single oncogene or tumor suppressor. For example, by joining two heterologous chromosomal fragments, translocation can generate a new oncogene, such as the formation of *BCR-ABL* by t(9:22) which drives chronic myeloid leukemia (CML).^28,29^ Translocations can also affect expression of an existing proto-oncogene, such as overexpression of c-MYC by t(8;14) in Burkitt lymphoma, which juxtaposes the coding region of *MYC* to the transcription regulatory elements of immunoglobulin heavy chain.^30^ Translocations can also disrupt a tumor suppressor gene, such as the disruption of *VHL* by germline t(1;3)(p36;p25) in the cancer-prone Von Hippel-Lindau disease.^31^

However, how chromosome translocation contributes to cancer is less clear when its biological consequences cannot be attributed to a single gene. Because of genome rearrangements that potentially affect hundreds or even thousands of genes, it is challenging to investigate the impact of these translocations on tumorigenesis. With recent advances in genomic editing technology, CRISPR/Cas9 and other similar tools had been quickly applied to induce translocations relevant to human cancers such as Ewing sarcoma, anaplastic large cell lymphoma, acute myelogenous leukemia, and lung cancer.^32–39^

In this study, we demonstrated that t(1;19)(q10;p10), which leads to the 1p/19q co-deletion in oligodendrogliomas,^10,11^ may be induced by CRISPR/Cas9-mediated genomic editing in glioma cells. The t(1;19)(q10;p10) translocation is likely mediated by homologous recombination between the centromeric repeats of chromosomes 1 and 19, which are nearly 100% identical.^40^ Because of the technical challenges associated with targeting repetitive sequences, we chose to introduce DSBs in nonrepetitive DNA sequences close to the centromeres (ie, within the chromosome 1p10 and 19q10 regions) by CRISPR/Cas9. The translocation and subsequent 1p/19q co-deletion were then achieved by NHEJ-mediated DSB repair. Consistent with previous studies,^32–39^ our data indicate that the overall efficiencies of forming the 1q/19p and 1p/19q hybrid chromosomes are low, ranging from 0.1% to less than 2%, likely depending on the chromosomes involved and the cell lines used (Figures 2–5). While translocation induction is feasible, our data suggest that clonal expansion and selection of cancer cells carrying the hybrid chromosomes is challenging, similar to the observations on selecting cells that have undergone other induced translocations.^41^ The challenge is not simply due to the low translocation efficiency and is likely caused by strong cellular stresses exerted by translocation and the accompanied deletion of chromosome arms in these cell lines. Only those cells with proper genetic compositions may benefit from such a drastic genetic event and gain growth advantages over the rest of the cell population that result in expansion. Because the 1p/19q co-deletion is primarily observed in oligodendrogliomas^4,5^ and with additional mutations in *IDH*, *TERT* promoter, and other genes (eg, *FUBP1* or *CIC*),^12–14^ we hypothesize that a proper cell type (eg, oligodendrocyte progenitor cells and oligodendrocytes), a permissive cellular microenvironment, and the presence of coordinating mutations (eg, *IDH*, *TERT* promoter, and *FUBP1* or *CIC*) are required to confer growth advantages to cancer cells with the 1q/19p hybrid chromosome and the 1p/19q co-deletion. Therefore, a more biologically informative and clinically relevant oligodendroglioma model would be introducing the 1p/19q co-deletion and other coordinating mutations (eg, *TERT* promoter, and *FUBP1* or *CIC*) in *IDH* mutant cells of the oligodendrocytic lineage.

In summary, we have developed a robust CRISPR/Cas9-based approach to induce t(1;19)(q10;p10), which results in 1p/19q co-deletion, in HEK 293T and GBM cells. If induced in proper cell types and with coordinating mutations, the method established here could lead to the development of a genetically defined research model for oligodendrogliomas, which will facilitate glioma research and the discovery of potential cures.

## Supplementary Material

vdac131_suppl_Supplementary_Material
