# Corrigendum to: Generation of chromosome 1p/19q co-deletion by CRISPR/Cas9-guided genomic editing

**DOI:** 10.1093/noajnl/vdad005

**Published:** 2023-01-16

This is a corrigendum to: Chao Li, Zhong Liu, Xiaoxia Zhang, Huafeng Wang, Gregory K Friedman, Qiang Ding, Xinyang Zhao, Hu Li, Kitai Kim, Xi Yu, L Burt Nabors, Xiaosi Han, Rui Zhao, Generation of chromosome 1p/19q co-deletion by CRISPR/Cas9-guided genomic editing, Neuro-Oncology Advances, Volume 4, Issue 1, January-December 2022, vdac131, https://doi.org/10.1093/noajnl/vdac131.

In the originally published version of this manuscript, there were errors in several figures: Figure 1A should state "1p/19q” instead of "1q/19p.” Primer labels in figure 3A should feature "1p" and "19q" instead of "1q" and "19p." Top panel of Figure 3B should state "1p/19q" instead of "1q/19p." Figure 3C should state “1p/19q PCR” instead of “1q/19p PCR.” Figure 3D should state “sequencing by the 1p primer" instead of "1q prime.r.” Figure 3E should state “sequencing by the 19q primer" instead of "19p primer.” Top panel of Figure 5B should state “1p/19q” instead of “1q/19p.” These errors have now been corrected.

