# Supplementary material for: Generation of chromosome 1p/19q co-deletion by CRISPR/Cas9-guided genomic editing
Source: Neurooncol Adv. 2022 Aug 18;4(1):vdac131. doi: 10.1093/noajnl/vdac131 (PMC9547542; doi:10.1093/noajnl/vdac131)
Supplement: vdac131_suppl_Supplementary_Material [file vdac131_suppl_supplementary_material.docx]

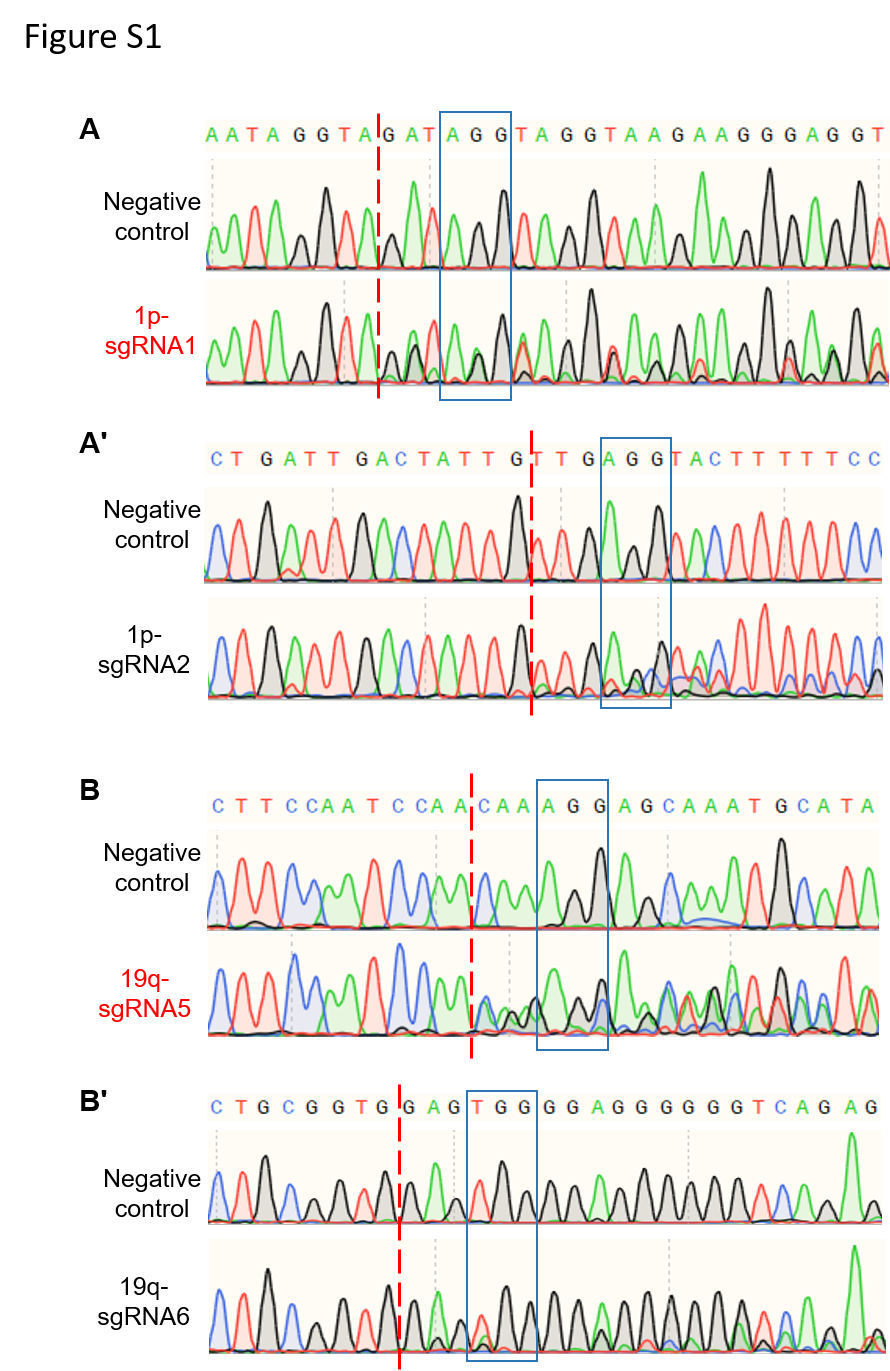


**Fig. S1. Evaluation of guide RNA (gRNA) efficiencies by automatic sequencing analysis.** **(A-A')** Evaluation of the two synthesized gRNAs targeting chromosome 1p. **(B-B')** Evaluation of the two synthesized gRNAs targeting chromosome 19q. Negative control, genomic DNA prepared from HEK 293T cells without CRISPR/Cas9 introduction; blue boxes, protospacer adjacent motif (PAM); red dashed lines, Cas9 cutting sites. 1p-sgRNA1 and 19q-sgRNA5 were selected for further analyses.


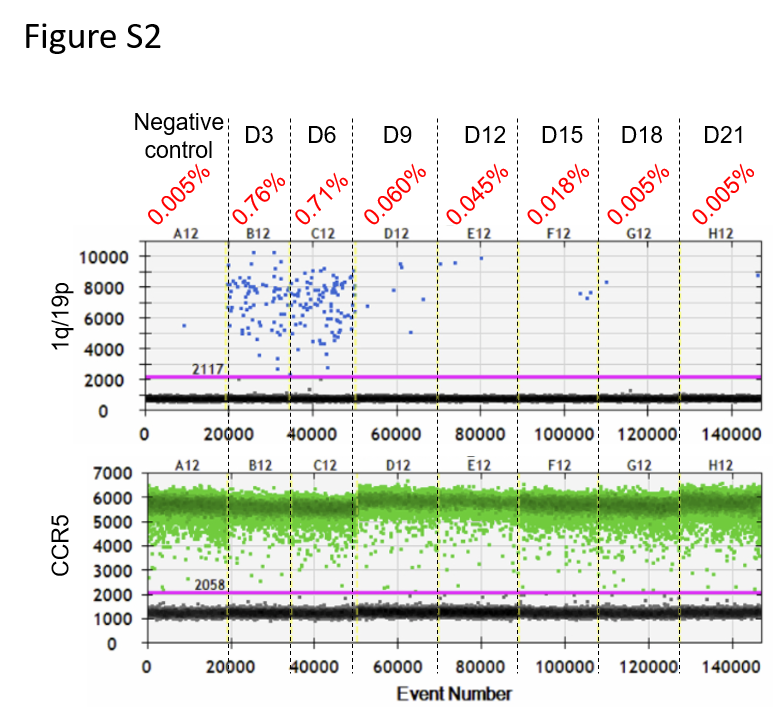


**Fig. S2. GBM cells carrying the hybrid 1q/19p chromosome are lost over time.** Droplet digital PCR (ddPCR) analysis of the 1q/19p product in LN-229 cells collected up to 21 days after CRISPR/Cas9 introduction. CCR5, internal control to normalize the copy number of input genomic DNA. The percentage shows the fraction of 1q/19p positive droplets of each sample.

## Table S1. Guide RNA sequences

| **gRNA** | **Sequence** |
| --- | --- |
| 1p-sgRNA1 | 5' – TTC CCT GTC AAT AGG TAG AT - 3' |
| 1p-sgRNA2 | 5' – TAA CTG ATT GAC TAT TGT TG - 3' |
| 19q-sgRNA5 | 5' – CAT CTC TTC CAA TCC AAC AA - 3' |
| 19q-sgRNA6 | 5' – TCA GCA ACT GCG GTG GAG TG - 3' |

## Table S2. Sequences of PCR primers and probes.

| **PCR Primers** | **Seqeunces** |
| --- | --- |
| 1p-f1 | 5' – TTC ACC AAC CAC AGA CAA GAA AG - 3' |
| 1p-f2 | 5' – CAT TTC AGC AGA ACC ACA ACA GA - 3' |
| 1p-r2 | 5' – GAG AGT TGA GCC TAG ACA GTT TGG - 3' |
| 19q-r5 | 5' – CAG ATA CCC TGC TAT GTT CTC TGA C - 3' |
| 19q-f6 | 5' – CCA TCA TCC TTG TCC CAC CTT - 3' |
| 19q-f7 | 5' – CCA TCC TCC CTT CCA ACC A - 3' |
| 19q-f10 | 5' – CCT TCA TTC ACG TGG CCA T - 3' |
| CCR5-f | 5' – TAC ATC GGA GCC CTG CCA- 3' |
| CCR5-r | 5' – ATG TCA GTC ATG CTC TTC AGC CT - 3' |
|  |  |
| **ddPCR probes** |  |
| 1q-probe-FAM | 5' – CAT AGC TAA AAG CAT GGC TTC TGG G - 3' |
| 19q-probe-FAM | 5' – TCA GCA ACT GCG GTG GAG TGG - 3' |
| CCR5-probe-HEX | 5' – CTC CGC TCT ACT CAC TGG TGT TCA TC- 3' |

**Table S3. Primer and probe combinations in ddPCR and nested PCR reactions.**

| Target | 1p/19q | 1q/19p |
| --- | --- | --- |
| ddPCR |  |  |
| PCR primers | 1p-f2 +19q-r5 | 19q-f7 +1p-r2 |
| Probe | 19q-probe-FAM | 1q-probe-FAM |
|  |  |  |
| Nested PCR and sequencing |  |  |
| PCR primers (1^st^ round) | 1p-f1 +19q-r5 | 19q-f10 +1p-r2 |
| PCR primers (2^nd^ round) | 1p-f2 +19q-r5 | 19q-f6 +1p-r2 |
| Sequencing primers | 1p-f2 , 19q-r5 | 19q-f6 , 1p-r2 |
